# Variant-Independent Association Between Clinical Symptoms and IgM/IgG Responses During the Transition from Pre-Omicron to Omicron SARS-CoV-2 Infections

**DOI:** 10.3390/microorganisms14051040

**Published:** 2026-05-04

**Authors:** Naim Che-Kamaruddin, Jefree Johari, Hasmawati Yahaya, Nurhafiza Zainal, Sazaly AbuBakar

**Affiliations:** 1 Tropical Infectious Diseases Research and Education Centre (TIDREC), Higher Institution Centre of Excellence (HICoE), Universiti Malaya, Kuala Lumpur 50603, Malaysia; jefree@um.edu.my (J.J.); hasmy@um.edu.my (H.Y.); 2 Institute for Advanced Studies, Universiti Malaya, Kuala Lumpur 50603, Malaysia; 3 Department of Medical Microbiology, Faculty of Medicine, Universiti Malaya, Kuala Lumpur 50603, Malaysia; nurhafizazainal@um.edu.my

**Keywords:** antibodies, cough, delta, omicron, Variants of Concerns (VOC)

## Abstract

Understanding how clinical symptoms relate to immune responses during major variant transitions remains important for informing post-pandemic surveillance and vaccination strategies. This study compared symptom patterns and SARS-CoV-2-specific anti-RBD IgM and anti-S1 IgG antibody responses among vaccinated individuals infected during the pre-Omicron and Omicron-dominant periods, representing a key phase in the evolution of SARS-CoV-2 population immunity. A retrospective analysis of 216 confirmed COVID-19 cases was performed by evaluating 11 predefined symptoms together with anti-RBD IgM and anti-S1 IgG levels measured at Day-14 after symptom onset, corresponding to the period when humoral antibody responses are detectable following SARS-CoV-2 infection. Participants with breakthrough infection during the Omicron-dominant period reported fewer symptoms overall compared to the pre-Omicron period, with a median of three versus four symptoms, respectively. Cough was the most common symptom during the Omicron period (82.1%), followed by sore throat (81.4%) and fever (78.6%). In contrast, loss of taste or smell was significantly more frequent in the pre-Omicron period (64.8% versus 22.9%, *p* < 0.05). IgG levels were significantly higher during the Omicron period than during the pre-Omicron period, increasing by 42.3%, reflecting enhanced antibody responses likely driven by repeated exposure. A consistent association between cough and elevated IgG levels was observed in both periods (*p* < 0.05), suggesting an association between symptom presentation and the magnitude of the early humoral response. These findings suggest that while clinical symptom profiles evolved across a major SARS-CoV-2 variant transition, certain symptom–antibody relationships remained consistent. Such associations may provide insight into how clinical manifestations relate to immune responses in populations with pre-existing immunity and may support interpretation of symptomatic infection during ongoing SARS-CoV-2 circulation.

## 1. Introduction

Coronavirus Disease 2019 (COVID-19) vaccines have been shown to effectively stimulate immune responses against severe acute respiratory syndrome coronavirus 2 (SARS-CoV-2), significantly reducing the risk of severe outcomes such as hospitalization and death [1,2,3]. As population immunity has increased through vaccination and infection, SARS-CoV-2 reinfections or breakthrough infections have been increasingly reported in individuals with pre-existing immune priming. Understanding how clinical symptoms relate to underlying immune responses during breakthrough infection remains important for interpreting disease presentation in the post-pandemic era, especially with highly transmissible variants such as Omicron [3].

COVID-19 symptoms can generally be categorized into systemic manifestations such as fever, headache, and muscle pain; respiratory manifestations such as cough, and shortness of breath; gastrointestinal symptoms including diarrhea and vomiting; and sensory symptoms, such as loss of taste/smell [4]. As SARS-CoV-2 has evolved and adapted to pre-existing immunity within the population, the patterns of symptoms and disease severity have also changed [3]. The Omicron variant, characterized by multiple spike protein mutations, demonstrates enhanced transmissibility and partial immune escape compared to earlier SARS-CoV-2 variants [5]. Despite reduced neutralization by pre-existing antibodies, infections during the Omicron period are associated with reduced clinical severity, including lower rates of hospitalization, intensive care unit (ICU) admissions, and mortality, as reported in previous studies [2,3]. This lower clinical impact is likely reflecting the contribution of memory immune responses generated through vaccination or prior infection [1].

Previous studies have shown that the severity of COVID-19 symptoms is associated with the kinetics of antibody responses. SARS-CoV-2-specific antibodies are typically detected earlier and at higher levels in individuals with more pronounced symptoms compared with those experiencing mild or asymptomatic infection [6]. Conversely, delayed antibody response has been associated with poorer clinical outcomes [7,8]. However, how symptom presentation relates to humoral immune responses during breakthrough infection in vaccinated populations, particularly across antigenically distinct variants, remains incompletely understood. While previous studies have reported associations between symptom severity and antibody responses, these investigations were largely conducted prior to the emergence of antigenically distinct variants or did not specifically examine vaccinated populations with pre-existing immunity. As a result, it remains unclear whether such symptom-immune relationships are consistent across different SARS-CoV-2 variants during breakthrough infection.

The present study therefore aimed to compare symptom patterns during the transition from pre-Omicron to Omicron SARS-CoV-2 circulation and evaluate their association with anti-Receptor-Binding-Domain immunoglobulin M (anti-RBD IgM) and anti-Spike protein S1 subunit immunoglobulin G (anti-S1 IgG) levels in vaccinated individuals experiencing breakthrough infection in the presence of vaccine-induced immunity. By examining symptom–antibody relationships across antigenically distinct variants, this study provides insight into how immune priming and viral evolution shape clinical presentation during respiratory viral infections.

Although the global burden of COVID-19 has declined, SARS-CoV-2 continues to circulate endemically, and the host determinants of symptomatic infection and immune activation remain important to understand. Analyzing infections that occurred during major variant transitions provides a valuable opportunity to examine how viral evolution interacts with pre-existing immunity in vaccinated populations. Identifying clinical features that consistently associate with humoral immune responses across antigenically distinct variants may provide insights into simplified indicators of immune activation. Such knowledge extends beyond SARS-CoV-2 by informing symptom-based surveillance approaches and improving preparedness for future emerging respiratory viral infections.

## 2. Material and Methods

### 2.1. Participant and Study Design

Informed consent was obtained from participants, and ethical approval was granted by the Universiti Malaya Medical Centre-Medical Research Ethics Committee (MREC-UMMC SID:2021226-9886).

This retrospective cross-sectional study analyzed a dataset comprising 216 individuals aged 15–65 years with confirmed SARS-CoV-2 breakthrough infection collected between March 2021 and April 2022 in Kuala Lumpur, Malaysia. Breakthrough infection was defined as a confirmed SARS-CoV-2 infection occurring in individuals who had completed COVID-19 vaccination prior to infection. SARS-CoV-2 infection was confirmed based on official reports provided by healthcare worker surveillance using polymerase chain reaction (PCR) or rapid test kits (RTK), which were documented in MySejahtera (the national digital platform used in Malaysia for COVID-19 vaccination and infection monitoring during the pandemic); no independent verification of test results was performed. Both PCR and RTK were used as part of routine clinical surveillance, and results were recorded in the national digital health system. While different diagnostic methods were used, all cases were confirmed according to national reporting criteria. COVID-19 vaccination data were also retrieved from digital vaccination certificates via the MySejahtera. Detailed vaccination history was recorded, including the number of doses and vaccine type (Pfizer-BioNTech BNT162b2, Pfizer Inc., New York, NY, USA / BioNTech SE, Mainz, Germany; Sinovac CoronaVac, Sinovac Biotech Ltd., Beijing, China; AstraZeneca ChAdOx1, AstraZeneca plc, Cambridge, UK). All vaccinations administered were based on the wild-type variant; no Omicron-adapted vaccines were administered to participants during the study period.

Additional demographic information, such as age, sex, and comorbidities, was obtained from the Case Report Forms (CRFs). Detailed protocols for the cohort immune response study have been previously described [9]. Briefly, participants were included if they had confirmed SARS-CoV-2 infection, had completed at least two doses of a COVID-19 primary vaccination series before the SARS-CoV-2 infection, had not received booster doses, provided a serum sample 14 days after symptom onset or test confirmation, and had complete vaccination and symptom records. Serum samples were separated and stored at −80 °C until analysis. Samples were processed according to standard laboratory procedures, and repeated freeze–thaw cycles were avoided to preserve antibody integrity. Individuals were excluded if they lacked vaccination documentation, did not provide Day-14 serum samples, had unverified test results, or had missing demographic or symptom data.

This analysis evaluated symptoms recorded during the acute infection phase together with antibody responses measured at Day-14 post-symptom onset or infection confirmation, corresponding to the period when humoral antibody responses typically reach detectable levels following SARS-CoV-2 exposure [10]. Symptoms were obtained at the time of recruitment from the CRFs using a predefined list of 11 commonly reported COVID-19 symptoms: fever, cough, sore throat, loss of taste and smell, shortness of breath, muscle pain, vomiting, diarrhea, pneumonia, respiratory distress, and headache [11]. Symptoms recorded in the CRFs referred to those present during the acute infection period represent infection-related clinical manifestations rather than vaccine-associated adverse events; symptom duration beyond Day-14 post-onset was not assessed.

Participants were categorized into two infection periods corresponding to major SARS-CoV-2 variant circulation phases in Malaysia: the pre-Omicron and Omicron-dominant periods. The pre-Omicron period was defined as infections occurring between March 2021 and December 2021, while the Omicron-dominant period was defined as January to April 2022 corresponding to the timeframe during which genomic surveillance data indicated that Omicron accounted for ≥75% of reported COVID-19 cases in Malaysia, based on publicly available national and global variant tracking data [12,13]. The pre-Omicron and Omicron-dominant periods consisted of independent participants, with no individuals appearing in both periods.

### 2.2. SARS-CoV-2 IgM and IgG Measurements

**Figure 1:**
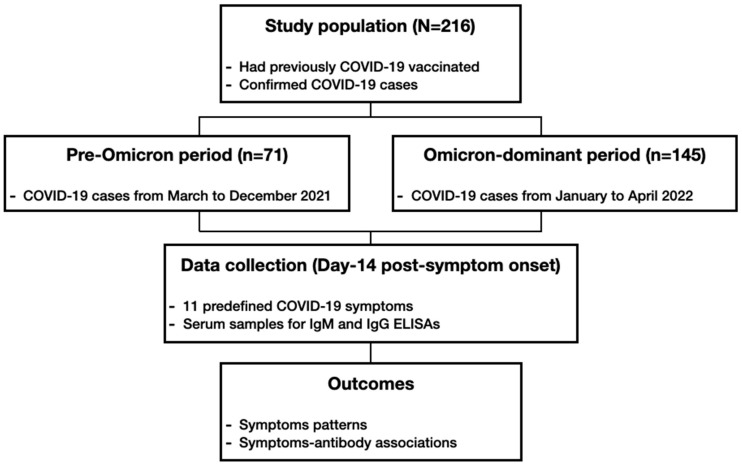
Schematic overview of the study design. The diagram illustrates participant selection, grouping into pre-Omicron and Omicron-dominant periods, symptom data collection, antibody measurement, and the analysis.

Anti-SARS-CoV-2 RBD-specific IgM and S1-specific IgG levels were measured from serum samples using commercially available enzyme-linked immunosorbent assays (ELISAs). RBD-specific IgM was measured using the Anti-SARS-CoV-2 RBD IgM ELISA (WANTAI, Beijing Wantai Biological Pharmacy Enterprise Co., Ltd., Beijing, China), following the manufacturer’s instructions. The positivity threshold for IgM was defined as an optical density (OD) value of 0.105. S1-specific IgG levels were measured using the Anti-SARS-CoV-2-S1 IgG ELISA (EUROIMMUN, Lubeck, Germany). Quantitative antibody concentrations were expressed as World Health Organization (WHO) international binding antibody units per mL (BAU/mL) using calibration standards provided by the manufacturer. The positivity cut-off value for IgG was defined as 35.2 BAU/mL. Both assays detect antibodies against the ancestral wild-type SARS-CoV-2 variant; variant-specific (e.g., Omicron) antibodies were not measured. A schematic overview of the study design and analysis workflow is illustrated in Figure 1.

### 2.3. Statistical Analyses

Participant demographic characteristics were summarized as categorical variables and presented as frequencies and percentages. Fisher’s exact test was used to compare variables between pre-Omicron and Omicron periods. To compare differences in COVID-19 symptoms during Omicron-dominant periods, vaccination type, and antibody levels among the participants, the Mann–Whitney U test was applied. A heatmap illustrating symptom correlations was generated through the Spearman correlation test to assess the simultaneous presence of different symptoms. IgG levels demonstrated a right-skewed distribution and were therefore log-transformed prior to analysis. Multivariable linear regression models were used to assess the association between symptoms and antibody levels, adjusting for age, gender, comorbidities, and vaccine type.

Statistical analyses were performed using R software version 2023.12.0+369. Statistical significance was determined at the 95% confidence interval, with *p*-values of 0.05 or less considered statistically significant.

## 3. Results

### 3.1. Overview of Participants’ Demographics

A total of 216 participants with documented SARS-CoV-2 infection were included in the analysis. The median age was 36 years, ranging from 15 to 65 years. Most participants were aged 31 to 40 years (41.3%, *n* = 87), followed by those aged ≤30 years (38%, *n* = 82), while 20.4% (*n* = 44) were aged 41 to 60 years, and 1.4% (*n* = 3) were over 61 years. Females comprised the majority of the cohort (62%, *n* = 134). All participants had completed a primary two-dose COVID-19 vaccination series prior to infection, with no booster doses administered. The most frequently administered vaccine was Pfizer-BioNTech (BNT162b2) (51.4%, *n* = 111) followed by AstraZeneca (ChAdOx1) (26.4%, *n* = 57) and Sinovac (CoronaVac) (22.2%, *n* = 48). The most commonly reported comorbidities were hypertension (7.4%, *n* = 16), asthma (6%, *n* = 13), and diabetes (4.6%, *n* = 10). No significant differences were observed between the pre-Omicron and Omicron periods with respect to age groups, sex, or comorbidities distribution (Table 1).

### 3.2. Symptom Frequency in COVID-19 Vaccinated Individuals During Pre-Omicron and Omicron-Dominant Periods

**Figure 2:**
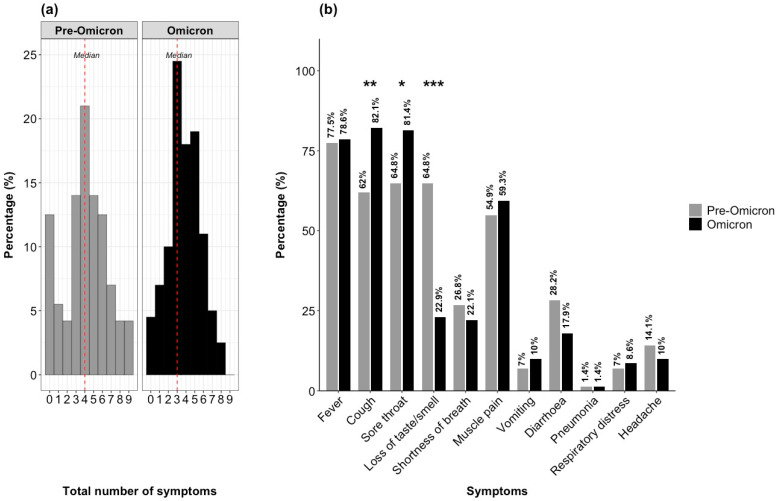
Percentage of participants by total number of symptoms and symptom prevalence. (**a**) Percentage of participants reporting varying total numbers of symptoms during the pre-Omicron and Omicron-dominant periods. The majority reported experiencing four symptoms in the pre-Omicron and three symptoms during the Omicron period. (**b**) Percentage of symptoms reported during the pre-Omicron and Omicron dominant period. Fever, cough, sore throat, and muscle pain were the most commonly reported symptoms in both periods, while loss of taste or smell was notably more prevalent during the pre-Omicron period than the Omicron period. Cough, sore throat, and loss of taste or smell were significantly different between the two periods. The *p*-values for symptom comparisons are indicated above the bar plot. Significant differences are marked as * *p* < 0.05, ** *p* < 0.01, *** *p* < 0.001.

Participants infected during the pre-Omicron period most commonly reported four concurrent symptoms (21%), whereas those infected during the Omicron period most commonly reported three symptoms (23%). The maximum number of symptoms reported was nine during the pre-Omicron period and eight in the Omicron period. Asymptomatic cases were more prevalent during the pre-Omicron period (13%) compared to the Omicron period (5%). The median number of symptoms reported was four in the pre-Omicron period and three in the Omicron period (Figure 2a).

The most prevalent symptoms during the pre-Omicron period were fever (77.5%), sore throat (64.3%), and loss of taste or smell (64.8%). In contrast, the most common symptoms during the Omicron period were cough (82.1%), sore throat (81.4%), and fever (78.6%). Loss of taste or smell was significantly more common during the pre-Omicron period compared to the Omicron period (64.8% vs. 22.9%), whereas cough and sore throat were more frequently reported during the Omicron period (Figure 2b).

### 3.3. Symptom Correlation Analysis During the Pre-Omicron and Omicron Periods

**Figure 3:**
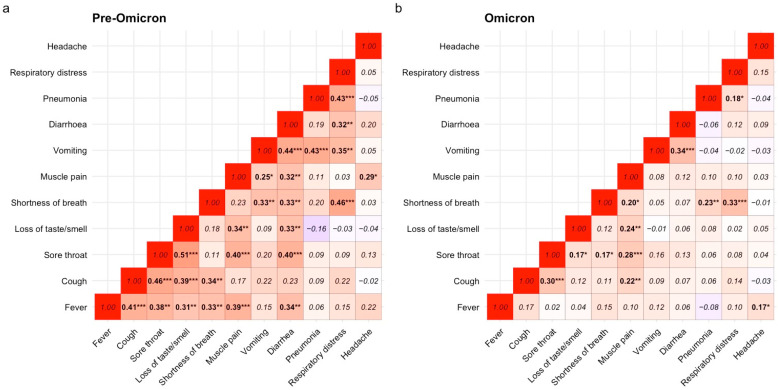
Heatmap of correlation matrix of symptoms in the pre-Omicron and Omicron-dominant periods. (**a**) Symptom correlation during pre-Omicron. The highest positive correlation was between loss of taste or smell and sore throat, which showed a moderate correlation (rs = 0.51, *p* < 0.001). (**b**) Symptom correlation during Omicron. The highest positive correlation was between diarrhea and vomiting, which showed a weak correlation (rs = 0.34, *p* < 0.001). The significance levels for each symptom pair are indicated by the asterisks, with their correlation coefficient displayed in the heatmap tiles. “rs” represents the Spearman correlation coefficient. Significant differences are marked as * *p* < 0.05, ** *p* < 0.01, *** *p* < 0.001.

Symptom co-occurrence patterns differed between the two variant periods (Figure 3). In the pre-Omicron period (Figure 3a), cough showed moderate positive correlations with shortness of breath (rs = 0.46, *p* < 0.001), fever (rs = 0.41, *p* < 0.01), and sore throat (rs = 0.51, *p* < 0.001), suggesting frequent co-occurrence of respiratory symptoms. A weak negative correlation was observed between loss of taste or smell and diarrhea (rs = −0.16, *p* < 0.05).

During the Omicron-dominant period (Figure 3b), cough remained positively correlated with fever (rs = 0.30, *p* < 0.001). Sore throat was positively correlated with loss of taste or smell (rs = 0.28, *p* < 0.001), while shortness of breath showed a moderate correlation with pneumonia (rs = 0.33, *p* < 0.001).

### 3.4. Symptom Frequency by COVID-19 Vaccination Type During the Omicron-Dominant Period

**Figure 4:**
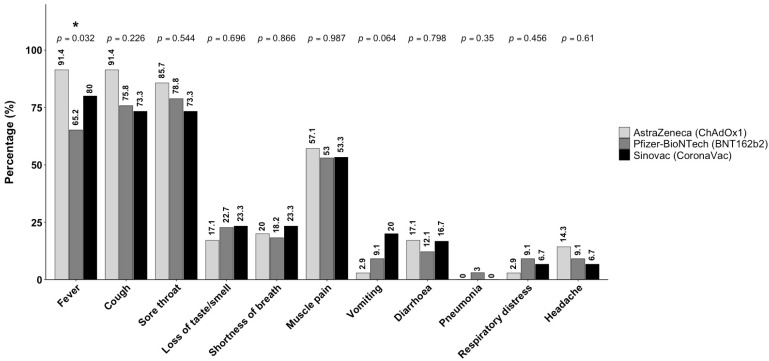
Symptom frequency by COVID-19 vaccination type during the Omicron-dominant period. A significantly higher prevalence of fever in individuals vaccinated with AstraZeneca-ChAdOx1 (91.4%) compared to Pfizer-BioNTech-BNT162b2 (65.2%) (*p* = 0.032) was observed. No significant differences were observed for other symptoms such as cough, sore throat, loss of taste or smell, shortness of breath, muscle pain, vomiting, diarrhea, pneumonia, respiratory distress, and headache across the different vaccination types, as determined by chi-square analysis. The *p*-values for symptom comparison are displayed above the bar plot. Significant differences are marked as * *p* < 0.05.

Symptom prevalence during the Omicron-dominant period varied according to vaccine type. Fever was significantly more common among individuals vaccinated with AstraZeneca (ChAdOx1) (91.4%) compared with those vaccinated with Pfizer-BioNTech (BNT162b2) (65.2%, *p* = 0.032). For cough and sore throat, symptom prevalence was high across all vaccination types, with no statistically significant differences observed (*p* = 0.226 and *p* = 0.544, respectively). Similarly, no significant differences were observed between vaccine types for other symptoms, including loss of taste or smell, shortness of breath, muscle pain, vomiting, diarrhea, pneumonia, respiratory distress, and headache (Figure 4).

### 3.5. Association of Symptoms with IgM and IgG Levels During the Pre-Omicron and Omicron Periods

Linear regression analysis was performed to evaluate associations between symptoms and antibody levels. During the pre-Omicron period, shortness of breath was significantly associated with higher IgM levels (Estimate = 0.36, *p* = 0.021). No other symptoms showed significant associations with IgM levels (Table 2). For IgG levels, cough (Estimate = 938.96, *p* = 0.031) and sore throat (Estimate = 956.81, *p* = 0.031) were significantly associated with higher antibody levels.

During the Omicron period, loss of taste or smell was significantly associated with higher IgM levels (Estimate = 0.08, *p* = 0.045). For IgG, cough remained significantly associated with higher IgG levels (Estimate = 546.32, *p* = 0.036), while sore throat showed a near-significant trend (*p* = 0.061). No significant associations were observed for other symptoms.

### 3.6. Multivariable Analysis of Factors Associated with IgG Levels

Multivariable linear regression analysis was performed to assess the association between symptoms and IgG levels while adjusting for age, gender, comorbidities, and vaccine type. After adjustment, cough remained significantly associated with higher IgG levels (adjusted β = 1.188, *p* < 0.001), indicating that this relationship is not explained by these factors. Age was also independently associated with IgG levels, whereas gender, comorbidities, and vaccine type were not significantly associated. These findings indicate that the association between cough and IgG levels persists after adjustment for measured confounding factors (Table 3).

### 3.7. Differences in IgM and IgG Levels Between Pre-Omicron and Omicron Periods

**Figure 5:**
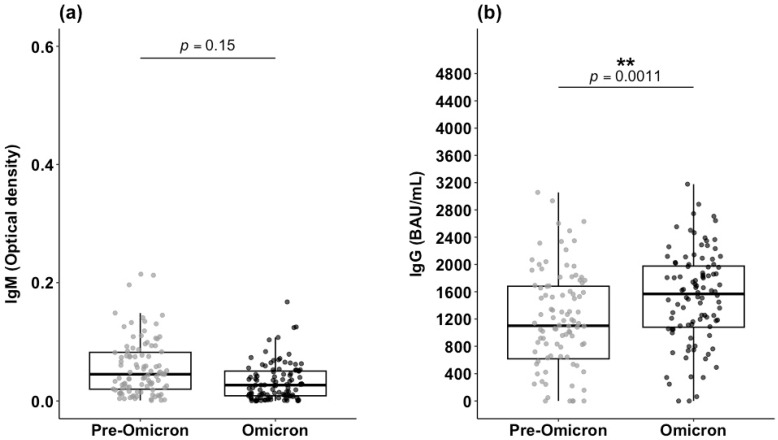
IgM and IgG levels during pre-Omicron and Omicron-dominant periods. (**a**) Anti-RBD IgM; (**b**) Anti-S IgG. Antibody levels for each period are shown as boxplots, with the top and bottom sides of the box representing upper and lower and quartiles, respectively, and the horizontal line splitting the box representing the median level. A Mann–Whitney U test was used to compare median differences between the periods, with the significance level displayed above the boxplot. ** Statistically significant.

Anti-RBD IgM levels measured at Day-14 post-symptom onset did not differ significantly between the pre-Omicron and Omicron periods (*p* = 0.15) (Figure 5a). However, IgG levels were significantly higher during the Omicron period compared with the pre-Omicron period (*p* = 0.001) (Figure 5b). The median IgG levels increased from 784.27 BAU/mL during the pre-Omicron period to 1359 BAU/mL during the Omicron period, representing a 42.3% increase (574.73 BAU/mL).

## 4. Discussion

This study examined symptom patterns and their association with humoral immune responses in vaccinated individuals infected during the transition from pre-Omicron to Omicron SARS-CoV-2 circulation. While clear shifts in symptom profiles were observed across variant periods, a consistent association between cough and higher IgG levels was identified in both groups. These findings suggest that certain symptom–immune relationships may be preserved despite antigenic variation and pre-existing immunity. In contrast to earlier studies conducted prior to the emergence of antigenically distinct variants, our results indicate that associations between clinical presentation and humoral immune responses may extend across variant transitions in vaccinated populations.

Although the dataset was collected during the pandemic period, it captures a unique and non-replicable epidemiological transition from pre-Omicron to Omicron SARS-CoV-2 circulation in a vaccinated population. This dataset provides an opportunity to examine how prior immune priming interacts with viral evolution to shape breakthrough infection presentations. These findings remain relevant in the context of endemic SARS-CoV-2 circulation and may inform understanding of host responses to other respiratory viral infections. Similar shifts in symptom profiles and immune responses across SARS-CoV-2 variants have been reported in other regions, including Europe, supporting the generalizability of these observations [11,14,15].

Significant changes in symptom prevalence were observed between the two variant periods. Loss of taste or smell, previously regarded as a hallmark symptom of COVID-19, was much more common during the pre-Omicron period, consistent with earlier reports showing strong involvement of neurologic and olfactory pathways. In contrast, respiratory symptoms such as cough and sore throat were significantly more frequently reported during the Omicron period. These observations are consistent with previous reports demonstrating reduced prevalence of loss of taste/smell during Omicron infections compared with earlier variants [14,15]. The shift in symptom profiles likely reflects differences in viral tropism and replication dynamics across variants. Earlier SARS-CoV-2 variants were shown to replicate efficiently in the lung parenchyma cells ex vivo [16], whereas the Omicron variant demonstrates enhanced replication in bronchial tissues [17,18]. These differences in tissue tropism may contribute to the increased prevalence of upper respiratory symptoms and the reduced occurrence of neurologic sensory symptoms observed during the Omicron period.

Beyond changes in individual symptoms, our correlation analysis revealed distinct patterns of symptom co-occurrence across variant periods. During the pre-Omicron period, cough frequently co-occurred with shortness of breath, fever, and sore throat, suggesting combined upper and lower respiratory tract involvement [16]. In contrast, symptom correlations during the Omicron period reflected a more consistent pattern with upper respiratory tract infection [18]. These findings suggest that symptom clustering may reflect differences in anatomical sites of viral replication and the magnitude of localized immune responses [19]. A key finding of this study is the consistent association between cough and elevated IgG levels across both variant periods, suggesting that certain clinical manifestations may be associated with the magnitude of the humoral immune response. Individuals reporting cough exhibited significantly higher IgG levels at Day-14 post-symptom onset in both the pre-Omicron and Omicron periods. Similar associations between respiratory symptoms and higher antibody responses have been reported in previous studies, where patients with more pronounced symptoms exhibited stronger humoral immune responses [6,7,8].

The immune responses elicited by different types of COVID-19 vaccines vary, which may influence the clinical manifestations observed during breakthrough infections [3,20]. In the present study, fever was significantly less common among individuals previously vaccinated with BNT162b2 compared with individuals who had received CoronaVac and ChAdOx1. This observation is consistent with previous studies reporting that recipients of mRNA vaccines, such as BNT162b2 and Moderna, generally exhibited milder symptoms during breakthrough infection compared with individuals vaccinated with viral vector vaccines such as ChAdOx1 [20]. The lower prevalence of fever among mRNA vaccine recipients may reflect more effective control of viral replication, which subsequently could modulate cytokine responses and reduce systemic inflammatory symptoms [21,22]. Although vaccination effectively reduces the severity and duration of COVID-19 symptoms, waning immunity and declining vaccine effectiveness over time can increase the likelihood of breakthrough infections [23].

In addition to differences in symptom patterns, the present study observed significantly higher IgG levels during the Omicron period compared with the pre-Omicron period at Day-14 post-symptom onset. The higher antibody levels observed during the Omicron period may reflect the cumulative effects of vaccination and repeated antigen exposure within the population. Omicron contains multiple mutations in the spike protein that allow partial escape from pre-existing antibodies while still stimulating strong secondary immune responses in vaccinated individuals [12]. Exposure to antigenically distinct SARS-CoV-2 variants such as Omicron may therefore activate memory B cells generated through prior vaccination or infection, resulting in enhanced IgG production. These findings support the concept that population-level immune responses continue to evolve as SARS-CoV-2 introduces new antigenic variations.

A key observation of this study is the consistent association between cough and elevated IgG levels in both the pre-Omicron and Omicron periods. Similar associations between respiratory symptoms and stronger antibody responses have been reported previously, where symptoms such as cough and fever were linked to higher SARS-CoV-2-specific IgG and IgA levels, particularly among individuals with mild COVID-19 [24,25]. In the present study, cough was reported in 62% of cases during the pre-Omicron period and 82.1% during the Omicron period, which is comparable to earlier reports indicating that cough occurred in approximately 60–70% of infections during the initial phase of the COVID-19 pandemic [26].

Although the present study focused on acute symptoms recorded within 14 days post-infection and did not assess long-term outcomes, persistent cough has been reported as a feature of post-acute sequelae of SARS-CoV-2 infection, affecting up to 42.6% of patients two months after illness [27]. Nevertheless, cough is a common and non-specific respiratory symptom that can arise from both upper and lower respiratory tract inflammation, and its presence does not necessarily indicate lower respiratory tract disease. Previous study has demonstrated that individuals with more pronounced symptoms tend to develop stronger antibody responses [6]. The association between cough and higher IgG levels observed in the present study therefore may reflect greater immune activation during acute infection rather than serving as a direct marker of lower respiratory tract involvement. Consistent with this interpretation, other symptoms typically associated with lower respiratory tract disease, such as pneumonia or respiratory distress, were not significantly correlated with antibody levels. Higher viral loads have been shown to drive stronger immune activation and antibody production [28,29], whereas infections limited primarily to the upper respiratory tract may involve lower viral burdens and weaker systemic immune responses [30,31]. Taken together, the relationship between cough and IgG levels observed in this study likely reflects the magnitude of immune activation during acute infection rather than the anatomical site of viral replication.

The mechanisms underlying the observed associations were not directly assessed in this study. Factors such as viral load, cytokine responses, mucosal immunity, and cellular immune responses may contribute to the relationship between symptom presentation and humoral responses. In particular, respiratory symptoms such as cough may reflect localized mucosal immune activation, which may not directly correspond to systemic antibody levels. Therefore, the observed associations should be considered hypothesis-generating and interpreted as indirect indicators of immune activation rather than direct mechanistic evidence. Several limitations of this study should be considered. First, symptom data were restricted to a predefined list recorded once during recruitment, which limited the analysis to acute-phase symptoms and may have resulted in underreporting of atypical or delayed clinical manifestations. Second, antibody measurements were obtained at a single timepoint (Day-14 post-symptom onset), which prevents assessment of longitudinal antibody kinetics, peak responses, and durability of immunity. The temporal separation between symptom onset and antibody measurement may also limit the interpretation of direct relationships between clinical presentation and humoral responses. In addition, symptom data were collected retrospectively, which introduces the possibility of recall bias and may affect the accuracy of reported symptom profiles. Third, the serological assays used in this study measured antibodies against the ancestral SARS-CoV-2 spike protein and do not specifically measure variant-specific neutralizing responses. This limitation is particularly relevant for infections during the Omicron-dominant period, as Omicron harbors multiple mutations in the spike protein that can reduce antibody binding and neutralization. As a result, the measured IgM and IgG levels may not fully reflect functional immunity against the infecting variant. Therefore, the observed associations between symptoms and antibody levels should be interpreted as reflecting general humoral immune activation rather than variant-specific protective immunity. Fourth, individual-level viral load measurements and genomic sequencing data were not available, and variant classification in this study was based on population-level genomic surveillance data rather than individual-level viral sequencing; as such, misclassification of infecting variants at the individual level cannot be excluded. However, the use of defined dominant periods (≥75% prevalence) provides a reasonable approximation of circulating variants at the population level. This limitation restricts the ability to attribute observed differences in symptom patterns or immune responses to specific variants at the individual level, and comparisons between variant periods should therefore be interpreted as population-level trends. Fifth, data on time since vaccination and prior infection history were not available, which may influence antibody levels and immune responses; therefore, residual confounding from these factors cannot be excluded. Given the retrospective cross-sectional design, causal relationships between symptoms and antibody responses cannot be established, and the findings should be interpreted as associations rather than causal effects. Despite these limitations, this study provides insight into clinical–immunological relationships that may remain informative even as viral variants evolve, offering a framework for interpreting symptomatic infection during endemic SARS-CoV-2 circulation and other emerging respiratory viruses.

## 5. Conclusions

The present study evaluated symptom patterns and their association with SARS-CoV-2 IgM and IgG levels in vaccinated individuals infected during the pre-Omicron and Omicron-dominant periods. We observed shifts in clinical presentation, with loss of taste or smell predominating during the pre-Omicron period, while cough and sore throat became more common during the Omicron period. Cough consistently correlated with higher IgG levels, suggesting an association between acute symptom presentation and the magnitude of the early humoral immune response.

These findings suggest that certain symptom–immune relationships may remain relatively consistent despite ongoing viral evolution. Understanding such relationships may support interpretation of symptomatic infection in populations with pre-existing immunity and provide insights into host responses to other emerging respiratory viral infections. In this context, SARS-CoV-2 serves as a useful model for examining how evolving respiratory viruses interact with pre-existing immunity at the population level.

## Figures and Tables

**Table 1:** Participants’ demographics. The variables are presented as frequencies and percentages, stratified by their respective periods. *p*-values were derived from Fisher’s exact test to compare differences across variables.

Variable	Overall (*N* = 216)		Pre-Omicron (*N* = 71)		Omicron (*N* = 145)		*p*-Value
	Frequency, *n*	Percentage, %	Frequency, *n*	Percentage, %	Frequency, *n*	Percentage, %	
Age Group							0.5
≤30 years old	82	37.96	21	29.58	61	42.07	
31–40 years old	87	40.28	35	49.30	52	35.86	
41–60 years old	44	20.37	14	19.72	30	20.69	
≥61 years old	3	1.39	1	1.41	2	1.38	
Sex							>0.9
Female	134	62.04	44	61.97	90	62.07	
Male	82	37.96	27	38.03	55	37.93	
Comorbidity							
Asthma	13	6.02	3	4.23	10	6.90	0.5
Cancer	0	0.00	0	0.00	0	0.00	0.7
Chronic Kidney Disease	1	0.46	0	0.00	1	0.69	0.5
Diabetes	10	4.63	3	4.23	7	4.83	0.9
Hemoglobin Disorder	3	1.39	2	2.82	1	0.69	0.4
Hypertension	16	7.41	7	9.86	9	6.21	0.3
Ischemic Heart Disease	0	0.00	0	0.00	0	0.00	0.7
Immunocompromised	1	0.46	1	1.41	0	0.00	0.5
Liver Disease	1	0.46	1	1.41	0	0.00	0.4
Obesity	9	4.17	3	4.23	6	4.14	>0.9
Sum Comorbidity							0.5
0	171	79.17	54	76.06	117	80.69	
1	33	15.28	15	21.13	18	12.41	
2	11	5.09	2	2.82	9	6.21	
3	0	0.00	0	0.00	0	0.00	
4	1	0.46	0	0.00	1	0.69	
Primary COVID-19 Vaccine Type							<0.001
AstraZeneca (ChAdOx1)	57	26.39	15	21.13	42	28.97	
Pfizer-BioNTech (BNT162b2)	111	51.39	45	63.38	66	45.52	
Sinovac (CoronaVac)	48	22.22	11	15.49	37	25.52	

**Table 2:** Association of symptoms with IgM and IgG levels during the pre-Omicron and Omicron-dominant periods. Specific symptoms, such as cough and sore throat during the pre-Omicron period and cough and loss of taste or smell in the Omicron period, were significantly associated with higher IgG and IgM levels, respectively. Significant associations are highlighted in bold and indicated by asterisks. * Statistically significant.

Symptom	Estimate	Std. Error	t-Value	*p*-Value
***Pre-Omicron (IgM)***				
Fever	0.125	0.165	0.753	0.454
Cough	0.210	0.141	1.493	0.140
Sore Throat	−0.013	0.145	−0.089	0.929
Loss of Taste or Smell	0.147	0.144	1.021	0.311
**Shortness Breath**	**0.356**	**0.151**	**2.361**	0.021 *
Muscle Pain	0.092	0.139	0.663	0.510
Vomiting	0.352	0.268	1.317	0.192
Diarrhea	−0.003	0.154	−0.017	0.986
Pneumonia	1.062	0.574	1.848	0.069
Respiratory Distress	0.444	0.266	1.670	0.099
Headache	0.032	0.199	0.160	0.873
***Pre-Omicron (IgG)***				
Fever	515.362	510.105	1.010	0.316
**Cough**	**938.958**	**427.574**	**2.196**	0.031 *
**Sore Throat**	**956.813**	**434.503**	**2.202**	0.031 *
Loss of Taste or Smell	732.992	440.765	1.663	0.101
Shortness Breath	293.108	483.681	0.606	0.547
Muscle Pain	537.827	426.616	1.261	0.212
Vomiting	−763.623	834.086	−0.916	0.363
Diarrhea	−12.619	477.296	−0.026	0.979
Pneumonia	−778.226	1819.557	−0.428	0.670
Respiratory Distress	140.508	838.966	0.167	0.867
Headache	625.546	612.587	1.021	0.311
***Omicron (IgM)***				
Fever	0.029	0.040	0.732	0.465
Cough	0.001	0.042	0.012	0.991
Sore Throat	−0.000	0.042	−0.005	0.996
**Loss of Taste or Smell**	**0.077**	**0.038**	**2.019**	0.045 *
Shortness Breath	0.049	0.039	1.249	0.214
Muscle Pain	0.060	0.033	1.824	0.070
Vomiting	0.004	0.054	0.081	0.936
Diarrhea	−0.063	0.042	−1.501	0.136
Pneumonia	0.067	0.137	0.492	0.624
Respiratory Distress	0.010	0.058	0.180	0.857
Headache	−0.005	0.054	−0.090	0.929
***Omicron (IgG)***				
Fever	302.837	243.688	1.243	0.216
**Cough**	**546.316**	**258.384**	**2.114**	0.036 *
Sore Throat	481.513	255.295	1.886	0.061
Loss of Taste or Smell	374.381	237.323	1.578	0.117
Shortness Breath	199.447	241.570	0.826	0.410
Muscle Pain	154.367	204.237	0.756	0.451
Vomiting	−168.933	334.856	−0.504	0.615
Diarrhea	−210.558	261.923	−0.804	0.423
Pneumonia	511.363	846.212	0.604	0.547
Respiratory Distress	571.365	355.872	1.606	0.111
Headache	−169.809	334.853	−0.507	0.613

**Table 3:** Multivariable linear regression analysis of factors associated with log-transformed SARS-CoV-2 IgG levels at Day-14 post-symptom onset. Models were adjusted for age, gender, comorbidities, and vaccination type. Significant associations are highlighted in bold and indicated by asterisks. * Statistically significant.

Variable	Estimate	Std. Error	*p*-Value
**Cough**	**1.188**	**0.246**	<0.001 *
**Age**	**0.042**	**0.011**	<0.001 *
Sex	0.015	0.213	0.944
Comorbidity	0.376	0.633	0.554
Vaccination type	0.174	0.249	0.486

## Data Availability

Due to ethical restrictions, the supporting data cannot be made openly available. Data are available from the corresponding author upon reasonable request and approval of the ethics committee.
